# Two G protein-coupled receptors bind ceramides in atherosclerosis progression: targets for a new therapeutic strategy

**DOI:** 10.1038/s41392-025-02434-1

**Published:** 2025-10-15

**Authors:** Yue Han, Peter Illes, Yong Tang

**Affiliations:** 1 International Joint Research Centre on Purinergic Signalling/School of Health and Rehabilitation, Chengdu University of Traditional Chinese Medicine, Chengdu, China; 2 Rudolf Boehm Institute for Pharmacology and Toxicology, University of Leipzig, Leipzig, Germany; 3 Tianfu Jincheng Laboratory, Chengdu, China

**Keywords:** Cardiology, Target identification

A recent study published by Zhang et al.^1^ in Nature suggested that the endogenous ceramide receptors cysteinyl leukotriene receptor 2 (CYSLTR2) and the pyrimidinergic receptor P2Y6 (P2Y6R) are potential novel targets for the treatment of atherosclerosis and related cardiovascular conditions beyond mainstream cholesterol control strategies. This new finding paves the way for another milestone in the emerging innovative search for novel atherosclerosis therapies.

Atherosclerosis is the leading cause of severe cardiovascular events such as myocardial infarction and stroke. Current drug treatment strategies focus primarily on lowering low-density lipoprotein cholesterol (LDL-C). Although statins and other lipid-lowering medications reduce the risk of cardiovascular adverse events by approximately one-third, a significant number of patients still face “residual risk” and experience adverse cardiovascular events even when their lipid levels are within target ranges.^2^ This is particularly true for patients with chronic kidney disease (CKD), where the efficacy of cholesterol reduction in preventing cardiovascular diseases diminishes as renal function declines, nearly disappearing in patients with end-stage renal disease.^3^ New targets or strategies to combat atherosclerosis beyond cholesterol reduction are urgently needed.

Ceramides are a class of sphingolipids formed by the linkage of sphingosine chains with fatty acid chains of varying lengths through amide bonds and signal via NOD-like receptor pyrin domain 3 (NLRP3) activation.^4^ A recent study via cryo-electron microscopy revealed that a Gi-coupled ceramide receptor acted through N-formyl peptide receptor 2 (FPR2) to regulate thermogenesis in adipocytes.^5^ Previous clinical studies have shown that long-chain ceramides in circulation, such as C16:0, C18:0, and C24:1, can predict the risk of cardiovascular events and are thus referred to as “second cholesterol.” However, how long-chain ceramides in circulation exacerbate the development of atherosclerotic cardiovascular diseases and whether there are membrane receptors that mediate their pathogenic signaling have long been open questions in the scientific community.

In this study, the authors identified for the first time a non-orphan endogenous membrane GPCR (G protein-coupled receptor) for circulating long-chain ceramides such as CYSLTR2 and P2RY6. Upon recognition of ceramides by these receptors, Gq protein activation is triggered, leading to activation of the NLRP3 inflammasome, which causes cleavage of caspase-1 (encoded by Casp1) and production of interleukin-1β (IL-1β), both of which significantly exacerbate the formation of atherosclerotic plaques. They also reported that the concentration‒response curves of C16:0 ceramide activated CYSLTR2–Gq and P2RY6–Gq signaling. Knocking out these receptors in animal experiments effectively blocked the pathogenic effects of C16:0 ceramide without affecting lipid levels. Blocking both receptors simultaneously had an even more pronounced effect on reducing atherosclerotic plaque burden.

CKD is a significant accelerator of the progression of atherosclerosis. To investigate the role of ceramides in exacerbating atherosclerosis in CKD patients, the authors collected and analyzed plasma samples from CHD patients at different stages of renal function. These data demonstrated significant increases in ceramides (C16:0, C18:0, C20:0 and C24:1) in patients with coronary artery disease (CAD) and CKD compared with patients with CAD and normal renal function, confirming an independent correlation between ceramide levels and the severity of coronary lesions. Knocking out the receptors or using receptor antagonists effectively reversed the progression of atherosclerosis aggravated by kidney disease without interfering with cholesterol levels. These findings suggest that ceramides could serve as novel predictive biomarkers for CKD combined with CHD and that targeting their receptors may block disease progression.

Additionally, the authors successfully elucidated the structure of the “C16:0/C20:0 ceramide-CYSLTR2-Gq” complex, clarifying that ceramides bind to the receptor in a nonclassical, oblique insertion (probably allosteric) manner, which includes a conserved sphingosine chain binding channel and a fatty acid binding pocket that can change shape to accommodate fatty acids of different lengths, providing an important blueprint for future drug development targeting ceramide receptors.

**Fig. 1:**
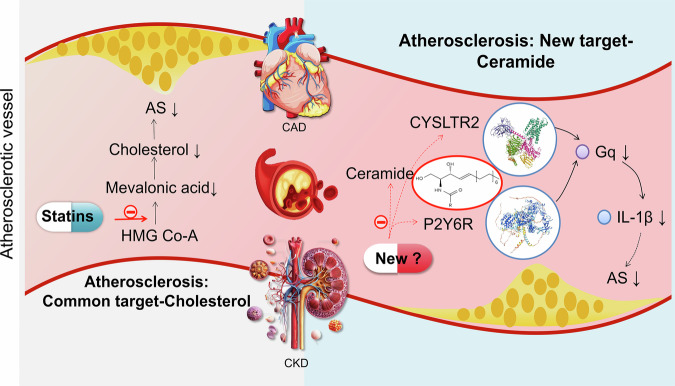
The ceramide receptors CYSLTR2 and P2Y6 are new targets for the treatment of atherosclerosis-related conditions (AS atherosclerosis CAD coronary artery disease, CKD chronic kidney disease, ↓ decrease), especially in nonresponders to statin treatment. Targeting CYSLTR2 and P2Y6Rs alleviates atherosclerosis without affecting the levels of ceramide and cholesterol and thereby prevents some adverse effects

This research finding is highly important, as it identifies the endogenous receptors CYSLTR2 and P2Y6R for ceramides for the first time, revealing the molecular mechanisms by which ceramides exacerbate atherosclerosis and CKD-related atherosclerosis through receptor activation and inflammasome activation. In addition, this discovery offers a new strategy for addressing the medical challenge of residual lipid risk. Targeting the ceramide receptors CYSLTR2 and P2Y6R might be a promising strategy for future atherosclerosis treatment, especially in the case of comorbid CKD, a condition in which one-third of AS patients exhibit no response to statins (Fig. 1). Interestingly, P2Y6Rs, which were previously thought to be specific for UDP binding, are also able to directly bind certain ceramides.
